# Investigation of elevation strength relative to upper-limb muscle mass in community-dwelling older adults with asymptomatic rotator cuff tears

**DOI:** 10.1016/j.jseint.2026.101795

**Published:** 2026-09-11

**Authors:** Hitoshi Koda, Yoshihiro Kai, Noriyuki Kida, Yuichiro Miura, Hideaki Fukushima, Tomoyuki Matsui, Hiromi Kondo, Minoru Takeshima, Toru Morihara

**Affiliations:** a Faculty of Arts and Sciences, Kyoto Institute of Technology, Sakyo-ku, Kyoto, Japan; b Department of Physical Therapy, Faculty of Health Science, Kyoto Tachibana University, Sakyo-ku, Kyoto, Japan; c Department of Rehabilitation, Marutamachi Rehabilitation Clinic, Sakyo-ku, Kyoto, Japan; d Department of Rehabilitation, Kumiyama Okamoto Hospital, Sakyo-ku, Kyoto, Japan; e Department of Orthopaedics, Ijinkai Takeda General Hospital, Sakyo-ku, Kyoto, Japan; f Department of Orthopaedics, Tanabe Central Hospital, Sakyo-ku, Kyoto, Japan; g Department of Orthopaedics, Marutamachi Rehabilitation Clinic, Sakyo-ku, Kyoto, Japan

**Keywords:** Asymptomatic rotator cuff tear, Shoulder elevation strength, Strength relative to upper-limb muscle mass, Subscapularis tendon, Upper-limb muscle mass, Older adults

## Abstract

**Background:**

Many rotator cuff tears (RCTs) in community-dwelling older adults remain asymptomatic. Here, we compared shoulder elevation strength relative to upper-limb muscle mass in this population.

**Methods:**

We analyzed 433 shoulders from 303 community-dwelling older adults without shoulder pain symptoms. RCTs were diagnosed using ultrasound imaging and classified into no tear (intact group), supraspinatus (SSP) or infraspinatus (ISP) tears (SSP/ISP group), and multiple tears, including the subscapularis (SSC) (SSP/ISP + SSC group). The relative strength per muscle mass and relative strength per body mass were calculated.

**Results:**

After applying the study criteria, 423 shoulders were analyzed: 358, 49, and 16 in the intact, SSP/ISP, and SSP/ISP + SSC groups, respectively. The mean strength-to-muscle mass ratios were 172.46 ± 55.87%, 150.41 ± 49.07%, and 107.47 ± 84.81% in the intact, SSP/ISP, and SSP/ISP + SSC groups, respectively. In the linear mixed-effects model, estimated relative strength per muscle mass tended to be lower in the SSP/ISP group than in the intact group; however, the difference was not statistically significant after Bonferroni adjustment. Shoulder elevation strength and relative strength per body mass did not differ significantly between the intact and SSP/ISP groups. No significant between-group difference was observed in upper-limb muscle mass.

**Conclusion:**

This study did not provide confirmatory evidence of an association between RCT classification and shoulder elevation strength relative to upper-limb muscle mass. However, relative strength per muscle mass may warrant further investigation as a complementary measure for identifying subtle functional differences that may not be apparent from absolute strength alone.

Rotator cuff tears (RCTs) are common in older adults and can cause shoulder pain, muscle weakness, and restricted range of motion.^16^ However, a substantial proportion of RCTs are asymptomatic and may be detected incidentally in population-based screening. In a community-based health checkup, Minagawa et al^21^ reported that 22.1% of middle-aged and older participants had full-thickness RCTs and 65.3% of these tears were asymptomatic. Similarly, Yamamoto et al^33^ reported that 65.4% of 283 shoulders with RCTs identified in community-dwelling older adults were asymptomatic, indicating that many RCTs in this population remain asymptomatic.

In symptomatic RCTs, pain during activity and pain-related avoidance behaviors are associated with reduced shoulder elevation strength.^20^ In contrast, findings regarding shoulder elevation strength in individuals with asymptomatic tears have been inconsistent. Decreased strength has been reported by those with extensive tear patterns, tears involving the subscapularis (SSC) tendon, and cases of muscle atrophy or fatty degeneration. Conversely, shoulder elevation strength is relatively preserved in many asymptomatic tears.^22^^,^^33^ As asymptomatic RCTs lack subjective symptoms, such as pain or movement restriction, tear-related functional impairment may remain sub-clinical in clinical practice.

Shoulder elevation relies on the coordinated function of the deltoid and rotator cuff.^6^ The deltoid is the main generator of elevation torque,^1^ whereas the rotator cuff stabilizes the humeral head by keeping it centered on the glenoid during elevation.^29^ Therefore, preserved cuff function allows the deltoid muscle mass to more directly contribute to elevation strength. However, degenerative rotator cuff changes that compromise tendon continuity or tension may reduce humeral head stability. Consequently, shoulder elevation may remain possible, but the deltoid may not generate elevation strength commensurate with its muscle mass.

Muscle strength is determined by multiple factors, including muscle quantity (skeletal muscle mass and cross-sectional area), neuromuscular activation, and efficiency of force transmission. Accordingly, muscle strength normalized to muscle mass, commonly expressed as the strength-to-muscle mass ratio, has been used as an index of relative strength.^4^ This ratio is associated with health-related and function-related outcomes in older adults, including sarcopenia.^15^^,^^24^^,^^32^ Building on this concept, we extended the strength relative to muscle mass to shoulder elevation by expressing elevation strength relative to upper-limb muscle mass. In shoulders with RCTs, disruption of tendon continuity or tension may reduce the ability of the rotator cuff to maintain the humeral head in a centered position on the glenoid, thereby limiting the extent to which upper-limb muscle mass is translated into elevation strength and potentially lowering the strength relative to muscle mass. Thus, elevation strength relative to upper-limb muscle mass may provide supplementary information about the functional consequences of rotator cuff impairment that cannot be obtained from absolute elevation strength. Therefore, by using data obtained from community-based orthopedic health examinations of older adults, we performed an exploratory comparison of elevation strength normalized to upper-limb muscle mass among asymptomatic shoulders according to the presence of RCTs and patterns of tendon involvement.

## Materials and methods

### Study design

This cross-sectional observational study was conducted as part of a community-based health examination program in Kyoto City, Kyoto, Japan.

### Participants

The participants were 303 community-dwelling older adults who underwent orthopedic health examinations. Both shoulders were evaluated, thus resulting in 606 shoulders as the units for analyses. The inclusion criteria were age ≥65 years, no shoulder pain, and an active shoulder range of motion of at least 150° in abduction and flexion. Shoulder pain status was assessed separately for each shoulder using a standardized face-to-face questionnaire with the question, “Have you experienced pain in your right/left shoulder in daily life during the past month?” The term “asymptomatic” in this study refers to the absence of subjective symptoms, not necessarily the absence of objective functional alterations. Of the 606 shoulders assessed, 433 met the inclusion criteria.

The exclusion criteria were prior upper extremity surgical intervention, neuromuscular disorders affecting upper-limb movements, and other pathologies affecting shoulder joint mobility. The exclusion criteria were applied at the shoulder level, and only the affected shoulder was excluded.

The study was conducted in accordance with the principles of the Declaration of Helsinki. The purpose, nature, and potential risks of the study were fully explained to the participants, who provided written informed consent prior to participation. This study was approved by the Institutional Review Board of Kyoto Tachibana University (approval numbers 23-46).

### Ultrasonographic assessment of rotator cuff tears

Bilateral shoulder ultrasonography was performed to assess the presence of RCTs. Examinations were conducted by an experienced orthopedic surgeon (>10 years of clinical experience) using a Noblus ultrasound system (Hitachi Aloka Medical, Tokyo, Japan). Previous studies have reported high interobserver agreement for the ultrasonographic detection and characterization of RCTs, including their classification as partial-thickness or full-thickness tears and identification of the tendons involved.^19^^,^^27^ The participants were examined in a sitting position with both arms relaxed at the sides and the shoulders positioned in neutral rotation. Using B-mode imaging, the supraspinatus (SSP), infraspinatus (ISP), and SSC tendons were evaluated in both the long-axis and short-axis views.^7^^,^^28^

The RCTs were diagnosed when discontinuity, flattening, or focal concavity or defects of the tendon surface were observed. For group classification, a shoulder was considered to have an RCT if either a partial-thickness or full-thickness tear was detected regardless of the tear size. Tear size, progression, and other detailed morphological characteristics were not quantified in this study.

Shoulders without tears were classified as intact. Shoulders with tears involving the SSP and/or ISP tendons were classified into the SSP/ISP group, whereas those with additional involvement of the SSC tendon were classified into the SSP/ISP + SSC group. Shoulders with isolated SSC tears were excluded from analyses.

### Primary outcomes

#### Shoulder elevation strength

Shoulder elevation strength was assessed using a previously validated functional elevation strength test for older adults.^14^ The participants sat upright in a chair, with the arm resting at the side for the start position, and were instructed to elevate the arm to at least 150° with the elbow fully extended while holding a dumbbell. This angle was selected because shoulder elevation of approximately 150° or greater is regarded as the highest range-of-motion category in commonly used shoulder function assessment scales such as the Japanese Orthopaedic Association and Constant scores. External loads ranging from 0.5 to 5.0 kg were tested in 0.5-kg increments, and the examiner adjusted the load based on task performance. The greatest load lifted to the target angle without compensatory trunk movements was recorded as functional shoulder elevation strength (kg). A rest interval of at least 30 seconds was provided between attempts, and testing continued only after confirming the absence of undue fatigue.

#### Skeletal muscle mass

Segmental skeletal muscle mass and body mass were estimated using a multifrequency bioelectrical impedance analyzer (MC-780A, TANITA Corporation, Tokyo, Japan). The participants stood barefoot on the platform and remained still while gripping the hand electrodes with both hands, as instructed by the manufacturer.^2^ A multifrequency current (330 μA) containing 5, 50, and 250 kHz components was applied via the 4 limbs to estimate body composition. The analyzer automatically outputs segmental skeletal muscle mass for each upper limb, and the right and left values (kg) were used for analyses. This value represents a regional estimate of the total skeletal muscle mass of the upper limb and does not provide muscle-specific measurements of the deltoid or rotator cuff muscles.

#### Relative strength per muscle mass

The relative strength per muscle mass was calculated for each shoulder as functional shoulder elevation strength (kg) divided by ipsilateral upper-limb skeletal muscle mass (kg), multiplied by 100 to express the value as a percentage (%).^4^ For comparison, the relative strength per body mass was calculated as functional shoulder elevation strength (kg) divided by body mass (kg), multiplied by 100.^12^

### Statistical analysis

The primary comparisons of shoulder elevation strength, upper-limb skeletal muscle mass, relative strength per muscle mass, relative strength per body mass, and basic characteristics among the intact rotator cuff, SSP/ISP tear, and SSP/ISP + SSC tear groups were performed using separate linear mixed-effects models. When a significant overall group effect was identified, pairwise comparisons of the estimated marginal means were performed with Bonferroni adjustment.

The exploratory sub-group analyses were performed to examine whether relative strength per muscle mass differed according to tear depth and tendon involvement pattern. Within the SSP/ISP tear group or the SSP/ISP + SSC tear group, partial-thickness and full-thickness tears were compared using the independent-samples *t*-tests. Because upper-limb strength and muscle mass may be influenced by limb dominance,^8^^,^^23^ a separate exploratory analysis comparing the dominant and non-dominant shoulders was performed using paired *t*-tests among participants with bilaterally intact rotator cuffs. All statistical analyses were performed using SPSS Statistics (version 28.0; IBM Corp., Armonk, NY, USA). All tests were 2-sided, and *P* < .05 was considered statistically significant.

## Results

### Participant characteristics

Of the 433 eligible shoulders, 9 with isolated SSC tears and 1 with missing data were excluded; thus, 423 shoulders were included in the analyses: 358 in the intact group, 49 in the SSP/ISP group, and 16 in the SSP/ISP + SSC group (Fig. 1). No significant group differences in age, height, or body mass were observed (Table I).

**Figure 1:**
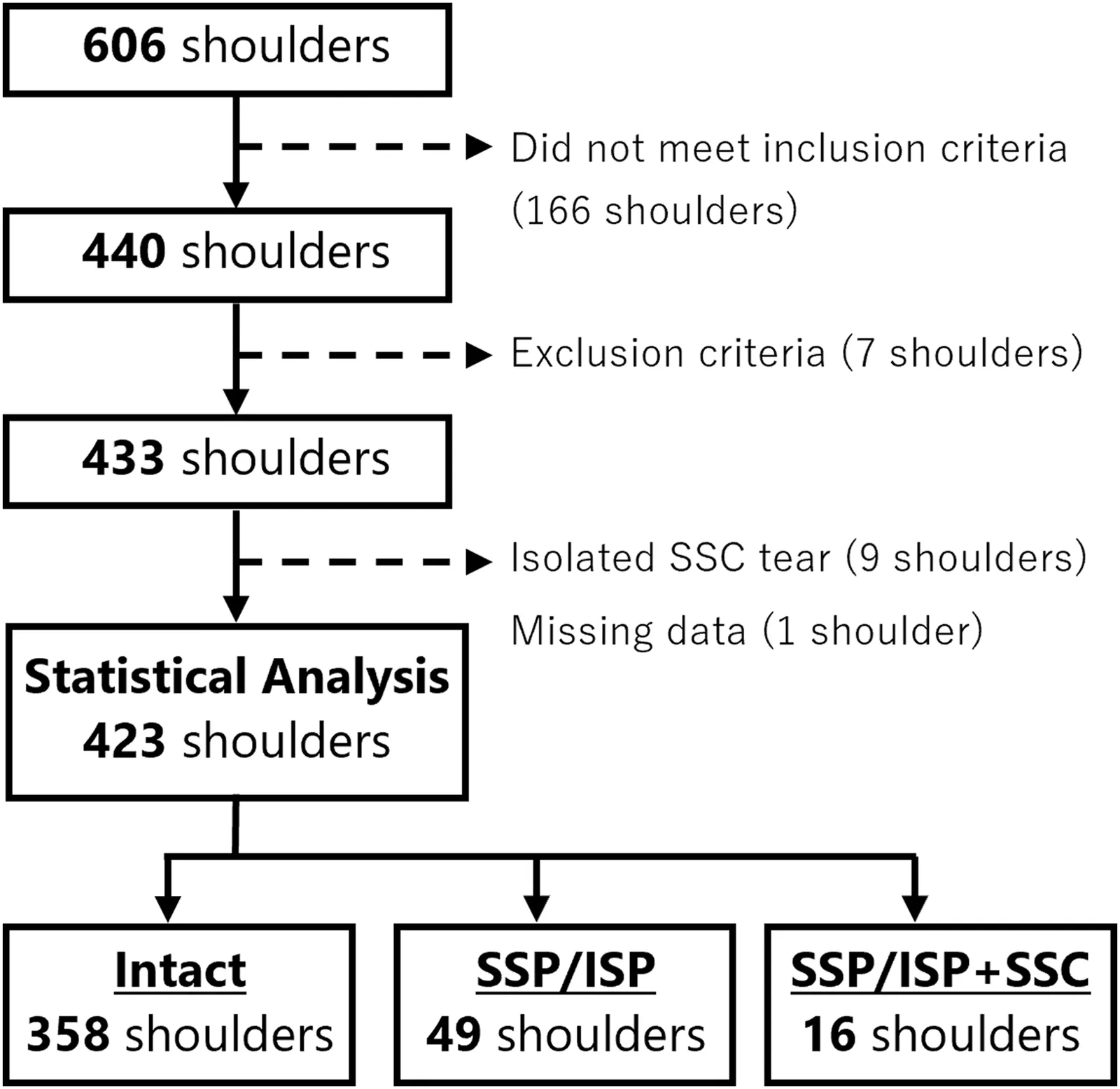
Flow diagram of shoulder selection and classification. Shoulders were screened at the shoulder level; cases of ineligible shoulders were excluded (including those with missing data and isolated SSC tears), and the remaining shoulders were classified into the intact, SSP/ISP, and SSP/ISP + SSC groups. *SSP*, supraspinatus; *ISP*, infraspinatus; *SSC*, subscapularis.

**Table I:** Baseline characteristics by rotator cuff tear pattern.

Characteristics	No tear group (358 shoulders)	SSP/ISP group (49 shoulders)	SSP/ISP + SSC group (16 shoulders)
Age (yr)	75.6 ± 5.0	76.3 ± 4.6	75.5 ± 4.1
Height (cm)	154.8 ± 7.5	155.8 ± 7.3	156.4 ± 9.1
Body mass (kg)	52.2 ± 8.0	55.3 ± 9.0	53.5 ± 8.4

*SSP*, supraspinatus; *ISP*, infraspinatus; *SSC*, subscapularis.

Values are presented as mean ± standard deviation. The unit of analysis was the shoulder. There were no significant differences.

### Shoulder elevation strength and muscle mass of the upper limb

The mean shoulder elevation strength values were 2.82 ± 0.99 kg, 2.72 ± 1.10 kg, and 1.84 ± 1.46 kg in the intact, SSP/ISP, and SSP/ISP + SSC groups, respectively. A significant group difference in the estimated shoulder elevation strength was observed (F_2,283.4_ = 6.312, *P* = .002), with the lowest values in the SSP/ISP + SSC group (Table II). The post-hoc comparisons indicated lower strength in the SSP/ISP + SSC group than in the intact (*P* = .001) and SSP/ISP groups (*P* = .016). The mean muscle mass ratios were 1.67 ± 0.40 kg, 1.81 ± 0.45 kg, and 1.78 ± 0.42 kg in the intact, SSP/ISP, and SSP/ISP + SSC groups, respectively. The estimated upper-limb skeletal muscle mass did not differ among the groups (F_2,278.9_ = 1.583, *P* = .207).

**Table II:** Estimated marginal mean and 95% confidence interval of related outcomes by rotator cuff tear pattern.

Variable	No tear group^a^ (358 shoulders)	SSP/ISP group^b^ (49 shoulders)	SSP/ISP + SSC group^c^ (16 shoulders)	Post-hoc test
Shoulder elevation strength (kg)	2.81	2.71	1.84	a, b > c†
	[2.67–2.95]	[2.40–3.03]	[1.33–2.36]	
Upper-limb skeletal muscle mass (kg)	1.68	1.79	1.78	n.s.
	[1.62–1.73]	[1.66–1.91]	[1.58–1.98]	
Relative strength per muscle mass (%)	170.41	151.63	107.47	a, b > c† a > b∗
	[163.05–177.78]	[134.7–168.57]	[79.56–135.36]	
Relative strength per muscle mass (%)	5.38	4.96	3.65	a > c† b > c∗
	[5.13–5.62]	[4.40–5.52]	[2.73–4.57]	

*n.s*, not significant; *SSP*, supraspinatus; *ISP*, infraspinatus; *SSC*, subscapularis.

Values are presented as estimated marginal means [95% confidence intervals]. The unit of analysis was the shoulder.

Relative strength per muscle mass = (shoulder elevation strength/upper-limb skeletal muscle mass) × 100.

Relative strength per body mass = (shoulder elevation strength/body mass) × 100.

In the post-hoc comparisons, a, b, and c indicate the no tear group, SSP/ISP group, and SSP/ISP + SSC group, respectively.

∗ Indicates *P* < .05 after Bonferroni adjustment.

† Indicates *P* < .05 in the unadjusted post-hoc comparison.

### Relative strength per muscle mass and relative strength per body mass

The mean relative strength per muscle mass values were 172.46 ± 55.87%, 150.41 ± 49.07%, and 107.47 ± 84.81% in the intact, SSP/ISP, and SSP/ISP + SSC groups, respectively. A significant group difference in the estimated relative strength per muscle mass was observed (F_2,296.9_ = 10.457, *P* < .001). The post-hoc comparisons indicated lower strength in the SSP/ISP + SSC group than in the intact (*P* < .001) and SSP/ISP groups (*P* = .024). The SSP/ISP group showed a lower estimated relative strength per muscle mass than the intact group in the unadjusted exploratory comparison (unadjusted *P* = .046); however, this difference was not statistically significant after Bonferroni correction (adjusted *P* = .139).

The mean relative strength per body mass values were 5.43 ± 1.78%, 4.93 ± 1.79%, and 3.65 ± 2.92% in the intact, SSP/ISP, and SSP/ISP + SSC groups, respectively. The estimated relative strength per body mass differed across the groups (F_2,286.3_ = 6.857, *P* = .001). The post-hoc analyses showed a lower ratio in the SSP/ISP + SSC group than in the intact group (*P* = .001), whereas no significant differences were observed between the SSP/ISP group and intact (*P* = .536) and SSP/ISP groups (*P* = .052).

### Sub-group exploratory analysis comparing partial-thickness and full-thickness supraspinatus/infraspinatus tears

In the SSP/ISP tear group, 40 shoulders had partial-thickness SSP/ISP tears and 9 had full-thickness SSP/ISP tears. Significant differences were not observed in shoulder elevation strength, muscle mass, and strength-to-muscle mass ratio between those with partial-thickness and full-thickness tears (Table III).

**Table III:** Sub-group comparisons between partial-thickness and full-thickness tears.

SSP/ISP tear depth	Shoulder elevation strength (kg)	*P* value	Upper-limb skeletal muscle mass (kg)	*P* value	Relative strength per muscle mass (%)	*P* value
SSP/ISP group						
Partial-thickness (40 shoulders)	2.63 ± 1.01	.187	1.78 ± 0.42	0.293	148.38 ± 49.72	.547
Full-thickness (9 shoulders)	3.17 ± 1.44		1.95 ± 0.55		159.44 ± 47.82	
SSP/ISP + SSC group						
Partial-thickness (7 shoulders)	2.64 ± 1.25	.084	1.64 ± 0.49	0.270	161.92 ± 63.62	.032∗
Full-thickness (8 shoulders)	1.38 ± 1.36		1.90 ± .38		73.27 ± 77.41	

*SSP*, supraspinatus; *ISP*, infraspinatus; *SSC*, subscapularis.

Sub-group comparisons between partial-thickness and full-thickness tears. Values are presented as mean ± standard deviation. The unit of analysis was the shoulder. The *P* values indicate comparisons between partial-thickness and full-thickness SSP/ISP tears within each tear group. In the SSP/ISP + SSC tear group, the sub-group comparison included only shoulders with partial-thickness SSC tears. The shoulder with a partial-thickness SSP/ISP tear and a full-thickness SSC tear was not included in the table or statistical comparison because it represented a single case.

∗ *P* < .05.

In the SSP/ISP + SSC tear group, 7 shoulders had partial-thickness tears in both the SSP/ISP and SSC, 8 had full-thickness SSP/ISP tears with partial-thickness SSC tears, and 1 had a partial-thickness SSP/ISP tear with a full-thickness SSC tear. Shoulders with full-thickness SSP/ISP tears and partial-thickness SSC tears had significantly lower strength-to-muscle mass ratios than those with partial-thickness tears in both the SSP/ISP and SSC (*P* = .032). No significant difference in the upper-limb skeletal muscle mass was observed according to the SSP/ISP tear depth. The 1 shoulder with a partial-thickness SSP/ISP tear and a full-thickness SSC tear was not included in the statistical comparison because there was only 1 case; the shoulder retained active arm elevation but was unable to perform elevation under external loading with a dumbbell.

A separate exploratory dominant–non-dominant comparison showed no significant side-to-side difference in relative strength per muscle mass among participants with bilaterally intact rotator cuffs. Detailed results are provided in the Supplementary Material.

## Discussion

In this study, we explored differences in shoulder elevation strength relative to upper-limb muscle mass among pain-free community-dwelling older adults according to the presence and patterns of RCTs. Shoulder elevation strength and relative strength per body mass did not differ significantly between the intact and SSP/ISP tear groups. Although relative strength per muscle mass tended to be lower in the SSP/ISP tear group than in the intact group, the difference did not remain statistically significant after Bonferroni adjustment in the linear mixed-effects model. The consistently lower estimates across shoulders with greater tendon involvement, together with the nominally significant unadjusted comparison, suggest that a possible relationship between RCT patterns and relative strength per muscle mass cannot be excluded.

Previous studies reported that asymptomatic RCTs do not necessarily present with measurable shoulder weakness.^9^^,^^11^ Hinsley et al^9^ reported no association between the tear size and shoulder strength. Additionally, Ueda et al^30^ found that asymptomatic shoulders with SSP tears had significantly greater abduction and external rotation strength than symptomatic shoulders, suggesting that pain substantially contributes to the weakness observed in RCTs. Consistent with these reports, absolute shoulder elevation strength did not differ significantly between the intact and SSP/ISP tear groups in the present study. Similarly, the relative strength per body mass did not differ between these 2 groups, although lower values were observed in the SSP/ISP + SSC group than in the intact group. Kim et al^12^ reported that the strength-to-body mass ratio was lower in asymptomatic shoulders with massive tears than in intact shoulders, whereas no difference was observed in shoulders with small tears. Upper-limb skeletal muscle mass showed no significant differences among the groups. Although fatty degeneration and muscle atrophy progress over time in RCTs,^18^^,^^34^ the upper-limb muscle mass assessed in this study reflected total upper-limb lean mass and was not specific to rotator cuff muscles. Taken together, absolute strength, body-mass-normalized strength, and regional upper-limb muscle mass considered separately may be insufficient to characterize subtle functional differences among asymptomatic shoulders.

The relative strength per muscle mass was lower in the SSP/ISP tear group than in the intact group in the unadjusted pairwise comparison; however, this difference did not remain statistically significant after Bonferroni correction. Muscle strength depends on morphological factors, such as skeletal muscle mass and muscle cross-sectional area,^17^ and neural factors such as motor unit recruitment and firing rate.^26^^,^^31^ Auyeung et al^3^ reported that in community-dwelling older men, skeletal muscle mass decreased by 1.59% over 4 years, whereas grip strength decreased by 3.85% over 2 years, indicating that age-related declines in muscle strength can be more pronounced than those in muscle mass. Although the relative strength per muscle mass tended to be lower with advancing age, age did not differ across the groups in this cohort, suggesting that the observed group differences were unlikely to be explained by age alone. Moreover, pain-related inhibition is unlikely to explain these findings, because this study included only asymptomatic tears. One possible explanation is that impaired tendon integrity or tendon tension may alter humeral head stability and force transmission during shoulder elevation. However, in the present study, we did not directly evaluate these physiological mechanisms, and upper-limb muscle mass included muscles other than those directly responsible for shoulder elevation. The observed difference should not be interpreted as evidence of reduced physiological efficiency, although it may suggest lower shoulder elevation strength relative to muscle mass in individuals with asymptomatic SSP/ISP tears. Although the present findings did not establish a definitive between-group difference, relative strength per muscle mass may provide a complementary perspective for examining shoulder function that is not captured by absolute strength or body-mass-normalized strength alone.

In addition, the relative strength per muscle mass values decreased further with SSC involvement and was lower in the SSP/ISP + SSC group than in the SSP/ISP group. The SSC forms a transverse force coupled with the ISP and teres minor, contributing to glenohumeral stability by helping to compress and center the humeral head on the glenoid during shoulder elevation.^5^ Massive tears involving the SSC compromise humeral head centering, leading to shoulder weakness, restricted range of motion, and, in some cases, pseudoparalysis (an inability to actively elevate the arm despite preserved passive motion).^5^^,^^25^ Although the present cohort consisted of asymptomatic shoulders and humeral head kinematics and tendon tension were not directly assessed, the lower shoulder elevation strength observed in the SSP/ISP + SSC group could reflect greater disruption of humeral head stabilization and force transmission during elevation. These findings suggest that SSC involvement is associated with a further reduction in the relative strength per muscle mass during shoulder elevation in older adults with RCTs.

In the exploratory sub-group analysis for tear depth, shoulders with full-thickness SSP/ISP tears combined with partial-thickness SSC tears had significantly lower relative strength per muscle mass than those with partial-thickness tears in both the SSP/ISP and SSC within the SSP/ISP + SSC tear groups. The patients with partial-thickness RCTs have greater shoulder strength than those with full-thickness tears.^13^ Collin et al^5^ reported that active elevation was significantly decreased in patients with more extensive massive RCT patterns. However, only 16 shoulders were included in the SSP/ISP + SSC group, and further stratification by tear depth resulted in very small sub-groups. Consequently, the estimates are likely to be imprecise, and the analyses are vulnerable to types I and II errors. No significant difference in the relative strength per muscle mass was observed between partial-thickness and full-thickness tears in the SSP/ISP tear group, indicating that tear depth alone was not clearly associated with this measure in that group. Nevertheless, this exploratory finding does not establish that full-thickness SSP/ISP tearing or concomitant SSC involvement independently accounts for the lower values but raises the possibility that tear depth and patterns of tendon involvement may be associated with shoulder elevation strength relative to upper-limb muscle mass.

This study has a few limitations. RCTs were identified using ultrasonography during a community health examination. Because the ultrasound protocol was designed primarily to identify the presence of tears and the tendons involved, detailed structural characteristics of the tears, including tear dimensions, tendon retraction, muscle atrophy, fatty infiltration, and tendon quality, were not comprehensively evaluated. Although previous studies have reported that tear size is not necessarily associated with lower strength,^11^ larger or more severe tears may be associated with lower relative strength per muscle mass. Future studies incorporating detailed imaging assessments are needed to clarify this issue. Consequently, the observed differences in the relative strength per muscle mass may partly reflect unmeasured differences in structural tear severity rather than tendon involvement alone. Moreover, intraobserver and interobserver reliabilities of the ultrasonographic classification were not directly evaluated in the present study, although all examinations were performed by the experienced examiner using standardized diagnostic criteria. Finally, upper-limb skeletal muscle mass was estimated using bioelectrical impedance analysis because this method was feasible for use in a community-based health examination involving a large number of older adults. However, bioelectrical impedance analysis provides only an indirect regional estimate and includes muscles that do not directly contribute to shoulder elevation. Although most upper-limb muscle mass is composed of muscles surrounding the shoulder joint,^10^ we did not quantify muscle mass in specific muscles (the deltoid) using imaging modalities, such as magnetic resonance imaging.

Despite these limitations, this study showed that shoulders with RCTs exhibited a lower relative strength per muscle mass even in asymptomatic patients without pain or movement restrictions. These findings suggest that shoulder elevation strength relative to upper-limb muscle mass may provide complementary information about subtle functional differences in asymptomatic RCTs. Future longitudinal studies are needed to determine whether this metric is associated with subsequent symptom development and functional decline.

## Conclusion

In this study, we compared shoulder elevation strength relative to upper-limb muscle mass among pain-free community-dwelling older adults according to rotator cuff tendon involvement. Shoulder elevation strength and the relative strength per body mass did not differ significantly between shoulders with SSP/ISP tears and intact shoulders. After accounting for within-participant correlation and multiple comparisons, no statistically significant differences in relative strength per muscle mass were identified among the tear groups. Therefore, the present findings do not provide confirmatory evidence that RCT classification is associated with relative strength per muscle mass.

Nevertheless, the estimated relative strength per muscle mass was lower in the SSP/ISP tear group than in the intact group, and this difference was observed in the unadjusted exploratory comparison. Although this finding should be interpreted cautiously because it did not remain statistically significant after Bonferroni adjustment, the direction of the estimated difference may provide a hypothesis for future research. Larger studies with prospectively specified comparisons and more detailed assessments of tear severity and shoulder muscle morphology are needed to determine whether relative strength per muscle mass can serve as a complementary marker of subtle functional differences associated with asymptomatic rotator cuff involvement.

## Disclaimers:

Funding: This study was supported by JSPS KAKENHI Grant Number JP25K14340. The funding source had no role in the study design, data collection, data analysis, interpretation of the results, preparation or editing of the manuscript, or the decision to submit the manuscript for publication.

Conflicts of interest: The authors, their immediate families, and any research foundations with which they are affiliated have not received any financial payments or other benefits from any commercial entity related to the subject of this article.

## Declaration of generative AI and AI-assisted technologies in the writing process

During the preparation of this work, the authors used ChatGPT-5 (OpenAI, San Francisco, CA, USA) in order to assist with English language polishing of text originally drafted by the authors. The manuscript was subsequently professionally edited for English language by Editage. The tool was not used for data analysis, interpretation, or conclusion generation. After using this tool/service, the authors reviewed and edited the content as needed and take full responsibility for the content of the publication.
