# Supplementary material for: Investigation of elevation strength relative to upper-limb muscle mass in community-dwelling older adults with asymptomatic rotator cuff tears
Source: JSES Int. 2026 Sep 11;10(6):101795. doi: 10.1016/j.jseint.2026.101795 (PMC13753320; doi:10.1016/j.jseint.2026.101795)
Supplement: Supplementary file 1 [file mmc1.docx]

**Supplementary Material.**

**Dominant versus non-dominant side comparisons in participants with bilaterally intact rotator cuffs**

|  | Dominant side | | |  | Non-dominant side | | |  | p-value |  |
| --- | --- | --- | --- | --- | --- | --- | --- | --- | --- | --- |
| Shoulder elevation  strength (kg) | 2.88 | ± | 0.97 |  | 2.80 | ± | 0.96 |  | 0.032 | * |
| Upper-limb skeletal muscle mass (kg) | 1.68 | ± | 0.41 |  | 1.62 | ± | 0.40 |  | < 0.001 | * |
| Relative strength per muscle mass (%) | 174.96 | ± | 55.21 |  | 177.83 | ± | 57.75 |  | 0.248 |  |

Values are presented as mean ± standard deviation. A dominant versus non-dominant side comparison was performed for participants with bilaterally intact rotator cuffs (n = 139; 278 shoulders; 134 right-hand dominant and 5 left-hand dominant). Shoulder elevation strength and upper-limb skeletal muscle mass were higher on the dominant side than on the non-dominant side. No significant side-to-side difference was observed in the relative strength per muscle mass. * p < 0.05.
